# The interaction between polyphenol intake and genes (MC4R, Cav-1, and Cry1) related to body homeostasis and cardiometabolic risk factors in overweight and obese women: a cross-sectional study

**DOI:** 10.3389/fnut.2024.1410811

**Published:** 2024-07-22

**Authors:** Zahra Roumi, Atieh Mirzababaei, Faezeh Abaj, Soheila Davaneghi, Yasaman Aali, Khadijeh Mirzaei

**Affiliations:** ^1^Department of Nutrition, Science and Research Branch, Islamic Azad University, Tehran, Iran; ^2^Department of Community Nutrition, School of Nutritional Sciences and Dietetics, Tehran University of Medical Sciences (TUMS), Tehran, Iran; ^3^Department of Nutrition, Dietetics and Food, School of Clinical Sciences at Monash Health, Monash University, Clayton, VIC, Australia; ^4^MSC, School of Nutrition and Food Sciences, Tabriz University of Medical Sciences, Tabriz, Iran

**Keywords:** cardiometabolic risk factors, genetic risk score, homeostasis, obesity, polyphenols

## Abstract

**Background:**

Cardiovascular disease (CVD), which is an important global health challenge, is expanding. One of the main factors in the occurrence of CVD is a high genetic risk. The interaction between genetic risk in CVD and nutrition is debatable. Polyphenols are one of the important dietary components that may have a protective role in people who have a high genetic risk score (GRS) for cardiometabolic risk factors. This study, conducted in overweight and obese women, examines the interaction between polyphenol intake and specific genes (MC4r, Cav-1, and Cry1) related to maintaining body balance and their interaction with cardiometabolic risk factors.

**Methods:**

This cross-sectional study included 391 women who were overweight or obese, aged 18 to 48 years, with a body mass index (BMI) between 25 and 40 kg/m^2^. Body composition was measured using the InBody 770 scanner. Total dietary polyphenol intake (TDPI) was assessed with a validated 147-item food frequency questionnaire (FFQ), and polyphenol intakes were determined using the Phenol-Explorer database. Serum samples underwent biochemical tests. The Genetic Risk Score (GRS) was calculated based on the risk alleles of three genes: MC4r, Cav-1, and Cry1.

**Results:**

The mean ± standard deviation (SD) age and BMI of women were 36.67 (9.1) years and 30.98 (3.9) kg/m^2^, respectively. The high GRS and high TDPI group had a significant negative interaction with fasting blood glucose (FBS) (*p* = 0.01). Individuals who had a high GRS and a high phenolic acid intake were found to have a significant negative interaction with Triglyceride (*p* = 0.04). Similarly, individuals with high GRS and a high intake of flavonoids had a significant negative interaction with TG (*p* < 0.01) and a significant positive interaction with High-density lipoprotein (HDL) (*p* = 0.01) in the adjusted model.

**Conclusion:**

According to our findings, those with a high GRS may have a protective effect on cardiometabolic risk factors by consuming high amounts of polyphenols. Further studies will be necessary in the future to validate this association.

## Introduction

Cardiovascular disease (CVD), which is the leading cause of death and a global health challenge, is becoming more prevalent (1–3). It is projected that by 2030, the number of deaths from this disease will reach 23.6 million (4). The rate of CVD in women is reported to be 1 in every 3 women, with 45% of women over the age of 20 affected (5, 6). Cardiometabolic risk factors like obesity, high blood pressure, dyslipidemia, and inflammation play a role in the development of CVD (7, 8).

Genetic background plays an important role in CVD (9). CVD is greatly influenced by the genetic predisposition of individuals (10). Research has shown that having a genetic risk for developing cardiometabolic risk factors increases the likelihood of certain health problems, emphasizing the importance of genetic factors in understanding cardiometabolic diseases (11). A genetic risk score (GRS) is an estimate of an individual’s genetic predisposition to a specific outcome, such as disease susceptibility. The combination of multiple genetic markers enables the prediction of disease risk based on an individual’s genetic profile (12). Genes play a crucial role in maintaining body homeostasis by regulating various metabolic processes. The research suggests that gene expression is closely connected to metabolite homeostasis, influencing adaptations in response to environmental changes and influencing energy efficiency and product formation (13).

Genetic mutations, like the Melanocortin 4 receptor (MC4R) gene mutation, can lead to obesity (14). This gene is situated in the hypothalamus (15, 16) and its mutation may indirectly contribute to a higher risk of mortality from CVD (17). The MC4R gene (rs17782313) not only influences obesity but is also associated with other risk factors for CVD, including hypertension (HTN) (18). Inactivation of the MC4R gene has been shown to reduce blood pressure independently of obesity in previous studies (19).

CAV-1, also known as Caveolin-1, is a protein that has been related to different biological processes and diseases. Research has found that CAV-1 levels are increased in individuals with metabolic syndrome (20). Studies reported that CAV-1 might have a role in the impairment of endothelial function, which is a fundamental anomaly in the development of hypertension, atherosclerosis, and coronary artery disease (21).

Moreover, the presence of CAV-1 in the cells lining the blood vessels can be affected by various factors including green tea polyphenols. The presence of CAV-1 in individuals with metabolic syndrome, a disease linked to insulin resistance (IR), high blood glucose levels, hypertension, abnormal lipid levels, obesity, and increased WC, has been observed (20, 22).

The Cryptochrome 1 (Cry1) gene is a molecular clock gene that plays a role in generating circadian rhythms (23). Evidence suggests a potential association between CRY1 (rs10861688) polymorphism, obesity and related cardiovascular risk factors (24). Research has shown that CRY1 is associated with components of metabolic syndrome, such as hypertension and triglyceride (25) levels, as well as obesity and insulin resistance (IR) (26, 27). Furthermore, recent research has suggested that variations and genetic differences in the human genome, including different forms of the Cry1 gene, could have an effect on energy expenditure and body weight (28).

Nutrition is a factor that influences GRS on the incidence of cardiometabolic diseases (29). Polyphenols, chemical compounds present in plants such as fruits, vegetables, and tea, have been shown in studies to be effective in decreasing the risk factors associated with CVD (30, 31). Previous studies have discussed various types of polyphenols, including flavonoids, stilbenes, phenolic acids, and lignans (32). Numerous research studies have examined the potential benefits of polyphenols in preventing obesity, and there is evidence to suggest that plant polyphenols have the potential to be effective in this area (33). In a cohort study, researchers discovered that elevated levels of flavanones and lignans were correlated with adult body composition, including BMI and waist circumference (WC) (34). Furthermore, a separate study conducted on women showed that polyphenols were linked to decreased fasting blood sugar (FBS) and blood pressure levels. Moreover, there was a significant correlation between elevated levels of high-density lipoprotein (HDL) cholesterol (35, 36).

The GRS allows us to explore how various genes related to cardiometabolic diseases interact with dietary intake to influence cardiometabolic risk factors. In this study, we aim to examine how a high consumption of polyphenols affects cardiometabolic risk factors in individuals with a high GRS, to determine if consuming high levels of polyphenols is beneficial in improving these risk factors.

## Method

### Study population

In this cross-sectional study, 391 overweight or obese women, aged between 18 and 48 years and with a body mass index (BMI) between 25 and 40 kg/m^2^ participated. These women were selected from people who visited 20 different health centers in Tehran using random sampling. Individuals with a prior medical history of cardiovascular or thyroid disease, malignancies, liver or kidney diseases, types of diabetes, acute or chronic diseases, pregnancy, lactation, or menopause, adherence to a specific diet or weight loss supplements, consumption of glucose and lipid lowering drugs and blood pressure medications within the past year, and smoking were not included in the study. Before the study, all participants were required to sign a written informed consent form. The study protocol received ethics approval from the Human Ethics Committee of Tehran University of Medical Sciences, with the ethics number IR.TUMS.MEDICINE.REC.1402.636. The procedures were conducted in compliance with applicable guidelines and regulations.

### Evaluation of dietary intake

The participants’ nutritional status, including energy intake, macronutrients, and micronutrients, was assessed using the food frequency questionnaire (FFQ) consisting of 147 items. Previous studies have confirmed the validity of this questionnaire for the Iranian population (37). A trained nutritionist conducted interviews with women to complete this questionnaire. The data were then analyzed using version 7 of the NUTRITIONIST 4 software, after being converted to grams using household measure servings (38).

### Evaluation of dietary polyphenol intake (DPI)

The Phenol-Explorer database (www.phenol explorer.eu/contents) was used to gather data on the overall polyphenol content in various foods (39). The total polyphenol content was determined either through the Folin Ciocalteu assay or by calculating the sum of four main subgroups, which include flavonoids, phenolic acids, stilbenes, lignans, and other polyphenols.

### Measurement of anthropometric indicators

Participants’ height was measured using a Seca stadiometer with an accuracy of 0.1 cm, and their weight was measured with a Seca digital scale (Hamburg, Germany) with an accuracy of 0.1 kg. To measure these two indicators, the participants must be without shoes and in the lightest clothes. BMI was calculated from the ratio of weight (kg) to the square of height (m^2^). In addition, to measure abdominal obesity, WC in the smallest circumference and hip circumference (HC) in the largest circumference were measured with an accuracy of 0.1 cm (40). Waist-to-hip ratio (WHR) was also calculated.

### Assessment of body composition

The InBody 770 Scanner, a multi-frequency bioelectrical impedance analyzer, was used to measure body composition parameters such as the amount and proportion of visceral fat level (VFL) and obesity degree (3). The measurements were taken in the morning while participants were in a fasted state and wearing light clothing. Participants were instructed to refrain from exercising, carrying electrical devices, and to urinate before the analysis to ensure accuracy. Following the manufacturer’s instructions, participants stood on the scale barefoot and held the machine’s handles for 20 s, after which the results were printed (41).

### Biochemical assessments

To assess the levels of biochemical factors (such as glucose and lipids) in the participants, blood samples were collected after a period of fasting and the serum was separated using a centrifuge. The serum was then divided into smaller portions and stored at −80°C until it could be analyzed. All blood parameters were measured in the Bionanotechnology Laboratory of the Endocrine and Metabolism Research Institute of Tehran University of Medical Sciences and analyzed using an exclusive assay based on the instructions provided by the manufacturer. All calculations were performed using a package from Randox Laboratories (Hitachi 902). The GPO-PAP method was employed to determine the levels of TG, while enzymatic and clearance endpoint assays were utilized to measure the total cholesterol (TC) and HDL cholesterol, respectively, in this research (42). Alanine aminotransferase (4) and aspartate aminotransferase (AST) were measured via standard protocols.

### Measurement of genetic risk score (GRS)

The DNA was obtained from whole blood samples through salting out techniques (43). The quality and quantity of the extracted DNA were evaluated using 1% agarose gel and the Nanodrop 8000 Spectrophotometer, respectively. TaqMan Open Array was used to genotype single nucleotide polymorphisms (SNPs), including CAV-1 (rs3807992), Cry1 (rs2287161), and MC4R (rs17782313) (44). These SNPs have been associated with obesity-related traits in previous studies (45–47). The GRS was computed by summing up the scores of the three SNPs, which were coded as 0, 1, or 2 based on their association with higher BMI. The unweighted GRS ranges from 0 to 6, with higher scores indicating a greater genetic predisposition to high BMI (48).

### Measurement of blood pressure

Systolic blood pressure (SBP) and diastolic blood pressure (DBP) were measured using standard sphygmomanometer and cuff through auscultation. After each subject had sat for at least 5 min, two consecutive blood pressure measurements were taken. Systolic blood pressure (SBP) and diastolic blood pressure (DBP) were measured with a standard mercury sphygmomanometer using the first and fifth Korotkoff sounds, to within 2 mmHg. If the difference between the two systolic or diastolic blood pressures was more than 5 mmHg, an additional measurement was performed.

### Assessment of other variables

Trained nutritionists filled out a demographic questionnaire that consisted of information regarding job, education, marital, and economic status. The present study measured PA as a confounding variable using the International Physical Activity Questionnaire (IPAQ). The data obtained from this questionnaire was measured on a scale of metabolic hours per week (MET. h week^−1^) (49).

### Statistical analysis

In the present study, cardiometabolic risk factors were determined based on biochemical, anthropometric and body composition criteria. The statistical analyses were performed using the IBM SPSS Statistics 23 software, with a significance level of less than 0.05. The normality of the distribution of the quantitative data of the study was performed by the Kolmogorov–Smirnov test. In this study, qualitative variables (Marriage, Education, Job and Economic Status) were described as numbers/percentages and quantitative variables (demographic variables, anthropometric measurements, body composition, blood parameters and blood pressure) were described as mean ± standard deviation (SD). The characteristics of the study participants among the tertile of GRS were compared with ANOVA and the characteristics of the study participants among the total dietary polyphenols index (TDPI) were compared with the independent t-test. To eliminate any confounding outcomes, ANCOVA was utilized. Both the crude and adjusted models employed a generalized linear model (GLM) to evaluate the interactions between metabolic factors and GRS, phenolic acid, lignans, flavonoids, and polyphenol. The outcomes were adjusted for age, energy intake, and PA.

## Results

### Study population characteristic

Our study was conducted on 391 overweight or obese women. The mean (± SD) age, weight, BMI and WC of participants were 36.67 (9.1) years, 80.28 (11.05) kg, 30.98 (3.9) kg/m^2^ and 99.16 (9.42) cm, respectively. Most of the participants were married (70.8%) and had no academic education (51%). They were also in a Moderate economic situation (45.5%).

### Characteristics of the study participants among tertile of GRS

The baseline characteristics of the study participants were presented in Table 1, categorized based on tertiles of their GRS. According to the table, in crude model there were a significant difference in mean values among the GRS tertiles for weight (*p* = 0.03), height (*p* = 0.03), WC (*p* = 0.03), and WHR (*p* = 0.03). There was also a marginally significant difference for TG (*p* = 0.06) among the GRS tertiles. After adjusting for confounding factors such as age, PA, and energy intake, the VFL (*p* = 0.03), SBP (*p* = 0.01), FBS (*p* = 0.02), and LDL (*p* = 0.01) became significantly different among the GRS tertiles. Additionally, there was a significant difference in height (*p* = 0.005), WC (*p* = 0.04), and WHR (*p* = 0.02), and a marginally significant difference for weight (*p* = 0.06) and TC (*p* = 0.08) among the GRS tertiles.

**Table 1:** Characteristics of the study participants among tertile of genetic risk score (GRS).

Variables		GRS				
		Low risk <3 (*n* = 164)	Moderate risk (3&4) (*n* = 97)	High risk ≥5 (*n* = 130)	*p*-value	*p*-value*
**Demographic variables**						
Age (years)		36.02 ± 8.78	36.53 ± 8.15	36.08 ± 8.61	0.91	0.89
PA (MET-minutes/week)		1052.27 ± 1116.01	1353.96 ± 2742.28	1389.51 ± 2576.15	0.61	0.58
**Anthropometric measurements**						
Weight (kg)		79.31 ± 9.79	76.9903 ± 10.29312	82.47 ± 9.33	**0.03**	0.06
Height (cm)		162.46 ± 5.41	160.26 ± 5.93	161.41 ± 4.45	**0.03**	**0.005**
BMI (kg/m^2^)		29.98 ± 3.31	30.09 ± 3.5	31.44 ± 3.32	0.16	0.22
WC (cm)		97.39 ± 8.62	96.4 ± 8.56	101.42 ± 9.03	**0.03**	**0.04**
WHR		0.92 ± 0.05	0.92 ± 0.04	0.95 ± 0.05	**0.03**	**0.02**
**Body composition**						
VFL (cm^2^)		14.9 ± 3.15	15 ± 3.07	16.16 ± 2.98	0.19	**0.03**
OD (%)		139.37 ± 15.47	139.91 ± 16.32	146.25 ± 15.54	0.16	0.22
**Blood pressure**						
SBP (mmHg)		111.24 ± 12.06	111.26 ± 15.38	113.08 ± 14.57	0.83	**0.01**
DBP (mmHg)		77.14 ± 10.08	77.44 ± 9.51	77.58 ± 11.04	0.97	0.16
**Blood parameters**						
FBS (mg/dL)		87.32 ± 9.2	86.46 ± 10.32	88.7 ± 9.82	0.57	**0.02**
TC (mg/dL)		186.41 ± 33.3	182.95 ± 37.7	179.83 ± 35.46	0.68	0.08
TG (mg/dL)		120.83 ± 57.88	108.35 ± 53.01	136.91 ± 75.67	0.06	0.11
HDL (mg/dL)		47.35 ± 9.69	47.59 ± 11.62	46.87 ± 11.6	0.95	0.98
LDL (mg/dL)		96.96 ± 21.67	95.12 ± 25.51	90.45 ± 25.82	0.51	**0.01**
AST (IU/L)		17.51 ± 6.9	17.97 ± 8.08	17.91 ± 8.56	0.92	0.79
ALT (IU/L)		18.16 ± 14.02	18.77 ± 13.31	20.25 ± 13.68	0.8	0.53
**Qualitative variable N (%)**						
Marriage status	Single	29 (49.2)	25 (42.4)	5 (8.5)	0.16	0.18
	Married	69 (35.9)	96 (50)	27 (14.1)		
Education levels	Non academic	49 (76.8)	62 (88.4)	22 (34.9)	0.3	0.32
	Academic	49 (42.2)	57 (49.1)	10 (8.6)		
Job	Unemployed	52 (34.7)	77 (51.3)	21 (14)	0.16	0.18
	Employed	45 (46.9)	40 (41.7)	11 (11.5)		
Economic status	Poor	19 (34.5)	27 (49.1)	9 (16.4)	0.77	0.77
	Moderate	46 (39.3)	56 (47.9)	15 (12.8)		
	Good	28 (43.8)	30 (46.9)	6 (9.4)		

BMI, Body mass index; WC, waist circumference; WHR, waist height ratio; FBS, fasting blood sugar; Chol, Cholesterol; TG, Triglyceride; HDL, High density lipoprotein; LDL, Low density lipoprotein; SBP, Systolic Blood Pressure; DBP, Diastolic Blood Pressure; OD, Obesity degree; VFL, Visceral fat level; TC, total cholesterol.

Quantitative variable: Mean ± SD (Standard deviation), Qualitative variable: N (%) Number (Percentage).

*p*-values < 0.05 are in bold.

*p*-value calculated by analysis of variance (ANOVA).

*p-value was found by ANCOVA, and adjusted for age, IPAC and total energy intake.

### Characteristics of the study participants among intake of TDPI

In Table 2, the characteristics of the participants are compared based on receiving low and high TDPI. The results showed that in the crude model, there is a significant difference between the two groups (low and high TDPI) in terms of job (*p* = 0.02), However in the adjusted model (age, PA, and energy intake), in addition to job (*p* = 0.01), the participants also had significant differences in terms of FBS (*p* < 0.001), TC (*p* = 0.01), and LDL (*p* < 0.001). Also a marginal difference for WC (*p* = 0.08) and WHR (*p* = 0.09) between the low intake and high intake groups of TDPI.

**Table 2:** Characteristics of the study participants among intake of total dietary polyphenols index (TDPI).

Quantitative variables		TDPI			
		Low intake (*n* = 196)	High intake (*n* = 195)	*p*-value	*p*-value *
**Demographic variables**					
Age (years)		35.44 ± 8.58	36.77 ± 8.42	0.23	0.31
PA(MET-minutes/week)		1086.64 ± 2159.97	1292.74 ± 2061.70	0.44	0.21
**Anthropometric measurements**					
BMI (kg/m^2^)		30.6 ± 4.06	30.29 ± 3.4	0.53	0.43
Weight (kg)		80.51 ± 11.04	79.73 ± 10.56	0.48	0.21
Height (cm)		160.82 ± 6.31	161.25 ± 5.31	0.47	0.35
WC (cm)		98.17 ± 9.06	97.32 ± 9.24	0.48	0.08
WHR		0.93 ± 0.046	0.92 ± 0.05	0.45	0.09
**Body composition**					
VFL (cm^2^)		15.32 ± 3.3	15.06 ± 3.3	0.55	0.45
OD (%)		142.21 ± 18.98	140.88 ± 15.84	0.56	0.43
**Blood parameters**					
FBS (mg/dL)		88.19 ± 11.18	86.77 ± 8.4	0.27	**<0.001**
TC (mg/dL)		186.86 ± 40.29	182.19 ± 32.73	0.33	**0.01**
TG (mg/dL)		116.29 ± 56.32	118.61 ± 60.03	0.76	0.31
HDL (mg/dL)		46.63 ± 11.28	47.39 ± 10.35	0.59	0.73
LDL (mg/dL)		94.68 ± 24.53	95.55 ± 23.98	0.78	**<0.001**
AST (IU/L)		17.97 ± 6.37	17.68 ± 8.11	0.76	0.81
ALT (IU/L)		18.68 ± 10.33	19.38 ± 14.93	0.68	0.48
**Qualitative variable**					
Marriage status	Single	58 (52.7)	52 (47.3)	0.29	0.36
	Married	138 (49.1)	143 (50.9)		
Education	Nonacademic	97 (49.5)	107 (55.4)	0.26	0.14
	Academic	99 (50.5)	86 (44.6)		
Job	Unemployed	105 (46.3)	122 (53.7)	**0.02**	**0.01**
	Employed	90 (57)	68 (43)		
Economic status	Poor	42 (47.7)	46 (52.3)	0.18	0.17
	Moderate	86 (47.3)	96 (52.7)		
	Good	62 (57.9)	45 (42.1)		

BMI, Body mass index; WC, waist circumference; WHR, waist height ratio; FBS, fasting blood sugar; Chol, Cholesterol; TG, Triglyceride; HDL, High density lipoprotein; LDL, Low-density lipoprotein; OD, Obesity degree; VFL, Visceral fat level; TC, total cholesterol.

TDPI Low intake < 2060.65 (mg/day), TDPI High intake ≥ 2060.65(mg/day).

*p*-values < 0.05 are in bold.

Quantitative variable: Mean ± SD (Standard deviation).

Qualitative variable N (%): N (%) Number (Percentage).

*p*-value calculated by analysis of independent sample *t*-test.

*p*-value *Adjusted for age, IPAC, and total energy intake calculated by analysis of covariance (ANCOVA).

### The interaction between GRS, TDPI, stilbenes, phenolic acid, lignans, flavonoids and polyphenol on cardiometabolic risk factors

The findings on the interaction between GRS, TDPI, and stilbenes on cardiometabolic risk factors are presented in Table 3. The crude and adjusted models showed a significant negative interaction between high GRS and high intake TDPI with FBS (crude: 95%CI = −20.39, −3.28, *p* < 0.001; adjusted: 95%CI = −19.95, −1.8, *p* = 0.01).

**Table 3:** The interaction between GRS, TDPI and stilbenes on cardiometabolic risk factors.

Variables	GRS	TDPI							Stilbenes						
		Low intake < 2060.65 (mg/day)	High intake						Low intake < 0.49 (mg/day)	High intake					
			Crude			Adjust				Crude			Adjust		
			β	95% CI	*p*	β	95% CI	*p*		β	95% CI	*p*	β	95% CI	*p*
**Anthropometric factors**															
BMI (kg/m^2^)	Low risk	Reference	Reference						Reference	Reference					
	Moderate risk	–	−3.04	−5.05 to −1.03	**<0.001**	−2.50	−4.55 to −0.46	**0.01**	–	0.48	−1.49 to 2.47	0.62	0.12	−1.89 to 2.14	0.9
	High risk	–	−1.81	−4.96 to 1.34	0.26	−1.79	−5.01 to 1.42	0.27	–	−1.16	−4.13 to 1.8	0.44	−0.43	−3.47 to 2.59	0.77
WC (cm)	Low risk	Reference	Reference						Reference	Reference					
	Moderate risk	–	−7.43	−12.53 to −2.33	**<0.001**	−7.45	−12.51 to −2.38	**<0.001**	–	3.27	−1.73 to 8.29	0.2	2.68	−2.36 to 7.73	0.29
	High risk	–	−5.29	−13.12 to 2.53	0.18	−5.59	−13.32 to 2.14	0.15	–	−2.35	−9.8 to 5.09	0.53	−1.21	−8.72 to 6.29	0.75
WHR	Low risk	Reference	Reference						Reference	Reference					
	Moderate risk	–	−0.01	−0.048 to 0.008	0.17	−0.02	−0.052 to 0.004	0.09	–	0.02	0.001–0.056	0.06	0.02	−0.005 to 0.05	0.1
	High risk	–	−0.02	−0.056 to 0.031	0.56	<0.001	−0.051 to 0.035	0.71	–	−0.01	−0.051 to 0.03	0.61	<0.001	−0.05 to 0.32	0.68
**Body composition**															
VFL (cm^2^)	low risk	Reference	Reference						Reference	Reference					
	Moderate risk	–	−2.11	−3.88 to −0.33	**0.02**	−2.06	−3.85 to −0.26	**0.02**	–	0.89	−1.66 to 1.83	0.92	−0.23	−2.02 to 1.55	0.79
	High risk	–	−1.02	−3.76 to 1.71	0.46	−0.82	−3.58 to 1.93	0.55	–	−0.96	−3.56 to 1.64	0.47	−0.31	−2.98 to 2.34	0.81
OD (%)	Low risk	Reference	Reference						Reference	Reference					
	Moderate risk	–	−6.70	−18.47 to 5.06	0.26	−6.77	−18.37 to 4.82	0.25	–	2.17	−9.32 to 13.67	0.71	−2.32	−13.78 to 9.13	0.69
	High risk	–	−8.52	−26.69 to 9.65	0.35	−9.56	−27.36 to 8.23	0.29	–	−7.73	−24.85 to 9.39	0.37	−4.72	−21.77 to 12.32	0.58
**Biochemical variables**															
FBS (mg/dL)	Low risk	Reference	Reference						Reference	Reference					
	Moderate risk	–	−9.16	−14.74 to −3.58	**<0.001**	−7.51	−13.38 to −1.63	**0.01**	–	3.06	−2.41 to 8.53	0.27	3.48	−2.29 to 9.17	0.24
	High risk	–	−11.83	−20.39 to −3.28	**<0.001**	−10.88	−19.95 to −1.8	**0.01**	–	2.58	−5.68 to 10.85	0.54	4.09	−4.66 to 12.84	0.36
TC (mg/dL)	Low risk	Reference	Reference						Reference	Reference					
	Moderate risk	–	−25.02	−46.51 to −3.54	0.22	−18.77	−40.94 to 3.39	0.09	–	1.51	−19.24 to 22.28	0.88	−3.86	−25.18 to 17.44	0.72
	High risk	–	−21.92	−54.89 to 11.04	0.19	−18.74	−52.97 to 15.49	0.28	–	3.88	−27.47 to 35.24	0.8	17.08	−15.44 to 49.62	0.3
TG (mg/dL)	Low risk	Reference	Reference						Reference	Reference					
	Moderate risk	–	−7.63	−44.58 to 29.23	0.68	0.45	−38.6 to 39.52	0.98	–	18.50	−16.65 to 53.67	0.3	14.71	−22.49 to 51.91	0.43
	High risk	–	−23.11	−81.29 to 35.06	0.43	−21.68	−83.49 to 40.12	0.49	–	4.36	−49.41 to 58.13	0.87	14.59	−42.95 to 72.15	0.61
HDL (mg/dL)	Low risk	Reference	Reference						Reference	Reference					
	Moderate risk	–	−1.42	−8.12 to 5.26	0.67	1.23	−5.8 to 8.26	0.73	–	0.30	−5.97 to 6.58	0.92	0.51	−6.12 to 7.14	0.88
	High risk	–	2.73	−7.53 to 13	0.6	1.09	−9.76 to 11.95	0.84	–	2.71	−6.7 to 12.26	0.56	4.77	−5.35 to 14.89	0.35
LDL (mg/dL)	Low risk	Reference	Reference						Reference	Reference					
	Moderate risk	–	−14.36	−28.75 to 0.028	**0.05**	−6.08	−21.04 to 8.87	0.42	–	6.25	−7.41 to 19.91	0.37	7.05	−7.12 to 21.24	0.33
	High risk	–	−20.03	−42.11 to 2.049	0.07	−21.10	−44.2 to 1.99	0.07	–	6.39	−14.24 to 27.03	0.54	12.57	−9.08 to 34.22	0.25
AST (IU/L)	Low risk	Reference	Reference						Reference	Reference					
	Moderate risk	–	−0.50	−5.05 to 4.038	0.82	0.83	−3.91 to 5.59	0.72	–	0.52	−3.8 to 4.86	0.81	1.94	−2.58 to 6.47	0.4
	High risk	–	−4.80	−11.78 to 2.16	0.17	−3.90	−11.25 to 3.43	0.29	–	−3.69	−10.51 to 2.57	0.23	−3.76	−10.68 to 3.14	0.28
ALT (IU/L)	Low risk	Reference	Reference						Reference	Reference					
	Moderate risk	–	−3.25	−11.27 to 4.76	0.42	−1.39	−9.92 to 7.14	0.74	–	0.64	−7.01 to 8.29	0.86	3.00	−5.14 to 11.14	0.47
	High risk	–	−5.93	−18.23 to 6.37	0.34	−4.02	−17.2 to 9.15	0.54	–	−2.71	−14.27 to 8.85	0.64	−0.96	−13.39 to 11.46	0.87

B, Standard Error; GRS, Genetic risk score; BMI, Body mass index; WC, waist circumference; WHR, waist height ratio; FBS, fasting blood sugar; TG, Triglyceride; LDL, Low density lipoprotein; HDL, High density lipoprotein; OD Obesity degree; VFL, Visceral fat level; TC, total cholesterol.

TDPI High intake ≥ 2060.65 (mg/day), Stilbenes High intake ≥ 0.49(mg/day).

*p*-values < 0.05 are in bold.

Adjust = adjusted for potential confounding factors including (age, IPAC and total energy intake).

Low Risk: 0,1,2 Risk alleles, Moderate Risk: 3,4 Risk allele, High Risk: 5,6 Risk allele.

**Figure 1:**
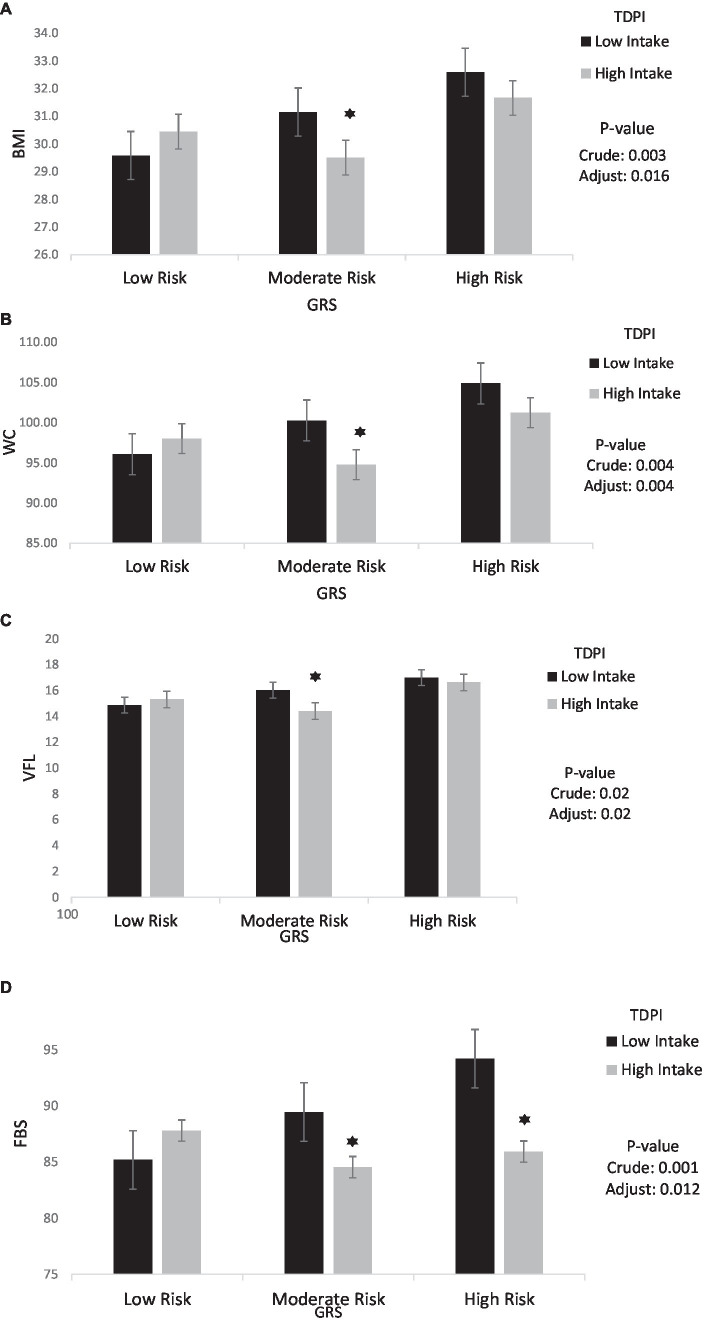
Interaction between TDPI and GRS on **(A)** BMI, **(B)** WC, **(C)** VFL, **(D)** FBS. The interaction between low and high intake of TDPI and GRS. Data shown are mean ± standard error of the mean. BMI, Body mass index; WC, Waist circumference; VFL, Visceral fat level; FBS, Fasting Blood Sugar; GRS, genetic risk score; TDPI, total dietary polyphenol intake. Adjust = adjusted for potential confounding factors including (age, IPAC and total energy intake). The asterisk (*) represents the *p*-value of the statistical test. The asterisk means that the *p*-value is less than 0.05.

The findings also showed that the significant negative interaction between moderate GRS and high intake TDPI with BMI (95%CI = −5.05, −1.03, *p* < 0.001), WC (95%CI = −12.53, −2.33, *p* < 0.001), VFL (95%CI = −3.88, −0.33, *p* = 0.02), FBS (95%CI = −20.39, −3.28, *p* < 0.001) and LDL (95% CI = −28.75, 0.028, *p* = 0.05) in the crude model. After adjusting for age, IPAC and total energy intake in model 1, the interaction between moderate GRS and high intake TDPI with BMI (95% CI = −4.55, −0.46, *p* = 0.01), WC (95% CI = −12.51, −2.38, *p* < 0.001), VFL (95% CI = −3.85, −0.26, *p* = 0.02) and FBS (95% CI = −13.38, −1.63, *p* = 0.01) remained negative. The interaction between TDPI and GRS on BMI, WC, VFL and FBS is shown in Figure 1.

Furthermore, there was no significant interaction found between moderate/high GRS and stilbenes with cardiometabolic risk factors in both the crude and adjusted models.

**Figure 2:**
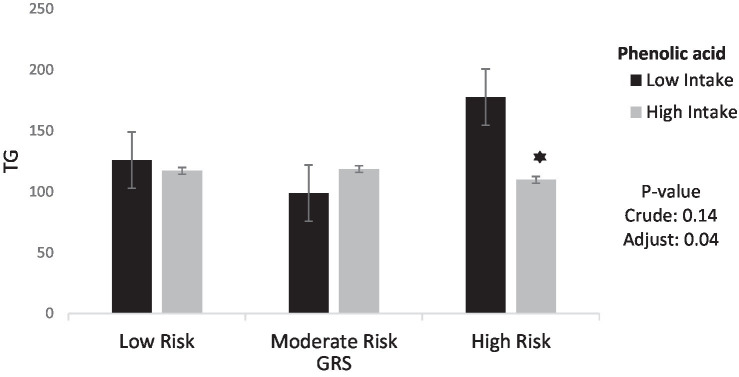
Interaction between phenolic acid and GRS on TG. The interaction between low and high intake of phenolic acid and GRS on Triglycerides. Data shown are mean ± standard error of the mean. TG, Triglycerides; GRS, genetic risk score. Adjust = adjusted for potential confounding factors including (age, IPAC and total energy intake). The asterisk (*) represents the *p*-value of the statistical test. The asterisk means that the *p*-value is less than 0.05.

The interaction between GRS, phenolic acid and lignans on cardiometabolic risk factors were presented in Table 4. In the crude model, a significant positive interaction was observed between high GRS and high intake phenolic acid on HDL (95%CI = 0.16, 19.23, *p* = 0.04) But in adjusted model, this interaction was not reported. However, a significant negative interaction was observed between high GRS and high intake phenolic acid on TG (95%CI = −115.66, −2.42, *p* = 0.04). Interaction between Phenolic acid and GRS on TG is shown in Figure 2.

**Table 4:** The interaction between GRS, phenolic acid and Lignans on cardiometabolic risk factors.

Variables	GRS	Phenolic acid							Lignans						
		Low intake < 55.95 (mg/day)	High intake						Low intake < 0.0065 (mg/day)	High intake					
			Crude			Adjust				Crude			Adjust		
			β	95% CI	*p*	β	95% CI	*p*		β	95% CI	*p*	β	95% CI	*p*
**Anthropometric factors**															
BMI (kg/m^2^)	Low risk	Reference	Reference						Reference	Reference					
	Moderate risk	–	−0.83	−2.83 to 1.16	0.41	−0.60	−2.62 to 1.41	0.55	–	−0.30	−2.29 to 1.69	0.76	0.48	−1.54 to 2.52	0.63
	High Risk	–	−0.59	−3.56 to 2.37	0.69	−1.26	−4.29 to 1.77	0.41	–	−1.08	−4.04 to 1.88	0.47	−0.60	−3.65 to 2.44	0.69
WC (cm)	Low Risk	Reference	Reference						Reference	Reference					
	Moderate Risk	–	0.33	−4.74 to 5.4	0.89	−0.06	−5.15 to 5.01	0.97	–	−0.69	−5.76 to 4.38	0.78	1.05	−4.03 to 6.15	0.68
	High Risk	–	2.09	−5.38 to 9.56	0.58	<0.001	−7.55 to 7.53	0.99	–	−3.52	−11 to 3.95	0.35	−2.20	−9.8 to 5.39	0.56
WHR	Low Risk	Reference	Reference						Reference	Reference					
	Moderate Risk	–	0.01	−0.01 to 0.04	0.35	0.01	−0.01 to 0.03	0.43	–	<0.001	−0.03 to 0.02	0.67	−2.24	−0.02 to 0.02	0.99
	High Risk	–	0.03	−0.009 to 0.072	0.12	0.02	−0.01 to 0.06	0.21	–	−0.01	−0.05 to 0.02	0.52	<0.001	−0.04 to 0.03	0.86
**Body composition**															
VFL (cm^2^)	Low risk	Reference	Reference						Reference	Reference					
	Moderate risk	–	−0.66	−2.42 to 1.09	0.45	−0.66	−2.44 to 1.12	0.46	–	0.57	−1.17 to 2.33	0.51	0.92	−0.86 to 2.72	0.31
	High risk	–	0.07	−2.51 to 2.67	0.95	−0.24	−2.89 to 2.41	0.85	–	−0.83	−3.42 to 1.76	0.39	0.03	−2.64 to 2.7	0.98
OD (%)	Low risk	Reference	Reference						Reference	Reference					
	Moderate risk	–	2.99	−8.57 to 14.56	0.61	0.25	−11.18 to 11.69	0.96	–	3.79	−7.58 to 15.54	0.5	5.08	−6.4 to 16.57	0.38
	High Risk	–	0.27	−16.82 to 17.37	0.97	−4.73	−21.75 to 12.28	0.58	–	−7.17	−24.29 to 9.94	0.41	−5.25	−22.42 to 11.9	0.54
Biochemical variables															
FBS (mg/dL)	Low risk	Reference	Reference						Reference	Reference					
	Moderate risk	–	0.45	−5.03 to 5.94	0.87	0.30	−5.45 to 6.05	0.91	–	1.19	−4.34 to 6.72	0.67	1.03	−4.78 to 6.84	0.72
	High risk	–	−2.71	−10.97 to 5.55	0.52	−3.39	−12.21 to 5.41	0.45	–	0.37	−7.96 to 8.7	0.93	0.41	−8.7 to 9.54	0.92
TC (mg/dL)	Low risk	Reference	Reference						Reference	Reference					
	Moderate risk	–	0.65	−20.11 to 21.42	0.95	−3.75	−24.96 to 17.45	0.72	–	16.88	−3.92 to 37.68	0.11	12.40	−8.99 to 33.81	0.25
	T3	–	−11.81	−43.07 to 19.44	0.45	−21.04	−53.54 to 11.45	0.2	–	4.59	−26.73 to 35.93	0.77	14.60	−18.97 to 48.18	0.39
TG (mg/dL)	Low Risk	Reference	Reference						Reference	Reference					
	Moderate risk	–	31.83	−2.74 to 66.41	0.07	28.60	−7.62 to 64.82	0.12	–	1.96	−33.53 to 37.45	0.91	−9.24	−46.61 to 28.13	0.62
	High risk	–	−39.54	−92.26 –13.17	0.14	−59.04	−115.66 to −2.42	**0.04**	–	16.72	−37.22 to 70.67	0.54	28.58	−30.54 to 87.7	0.34
HDL (mg/dL)	Low risk	Reference	Reference						Reference	Reference					
	Moderate risk	–	3.10	−3.23 to 9.44	0.33	2.87	−3.77 to 9.52	0.39	–	−0.13	−6.56 to 6.3	0.96	2.13	−4.6 to 8.87	0.53
	High risk	–	9.70	0.16–19.23	**0.04**	7.89	−2.3 to 18.08	0.12	–	1.85	−7.83 to 11.54	0.7	7.02	−3.55 to 17.59	0.19
LDL (mg/dL)	Low risk	Reference	Reference						Reference	Reference					
	Moderate risk	–	6.33	−7.48 to 20.14	0.36	9.11	−5.01 to 23.25	0.2	–	9.69	−4.22 to 23.61	0.17	11.26	−3.14 to 25.66	0.12
	High risk	–	−7.33	−28.12 to 13.45	0.48	−13.89	−35.54 to 7.76	0.2	–	0.19	−20.76 to 21.16	0.98	3.34	−19.24 to 25.9	0.77
AST (IU/L)	Low risk	Reference	Reference						Reference	Reference					
	Moderate risk	–	−2.93	−7.25 to 1.37	0.18	−2.68	−7.19 to 1.83	0.24	–	−2.01	−6.39 to 2.36	0.36	−0.54	−5.13 to 4.04	0.81
	High risk	–	−2.31	−8.8 to 4.17	0.48	−1.86	−8.78 to 5.05	0.59	–	−1.35	−7.95 to 5.23	0.68	2.03	−5.16 to 9.23	0.58
ALT (IU/L)	Low risk	Reference	Reference						Reference	Reference					
	Moderate risk	–	−6.83	−14.38 to 0.71	0.07	−5.45	−13.49 to 2.59	0.18	–	−7.23	−14.9 to 0.43	0.06	−4.59	−12.79 to 3.59	0.27
	High Risk	–	−0.28	−11.64 to 11.07	0.96	−0.12	−12.45 to 12.2	0.98	–	−3.70	−15.26 to 7.84	0.52	1.02	−11.83 to 13.87	0.87

B, Standard Error; GRS, Genetic risk score; BMI, Body mass index; WC, waist circumference; WHR, waist height ratio; FBS, fasting blood sugar; TG, Triglyceride; LDL, Low-density lipoprotein; HDL, High-density lipoprotein; OD, Obesity degree; VFL, Visceral fat level; TC, total cholesterol.

Phenolic acid High intake ≥ 55.95(mg/day), Lignans High intake ≥ 0.0065(mg/day).

*p*-values < 0.05 are in bold.

Adjust = adjusted for potential confounding factors including (age, IPAC and total energy intake).

Low Risk: 0,1,2 Risk alleles, Moderate Risk: 3,4 Risk allele, High Risk: 5,6 Risk allele.

Furthermore, there was no significant interaction found between moderate/high GRS and lignans with cardiometabolic risk factors in both the crude and adjusted models.

The interaction between GRS, flavonoids and polyphenol on cardiometabolic risk factors were presented in Table 5. The crude and adjusted models showed a significant negative interaction between high GRS and high intake flavonoids with TG (crude: 95%CI = −137.58, −31.2, *p* < 0.001; adjusted: 95%CI = −135.52, −22.69, *p* < 0.001). Also, a significant positive interaction was observed between high GRS and high intake flavonoids with HDL (95%CI = 2.47, 22.65, *p* = 0.01) in the adjusted model.

**Table 5:** The interaction between GRS, flavonoids and polyphenol on cardiometabolic risk factors.

Variables	GRS	Flavonoids							Polyphenol						
		Low intake < 81.55 (mg/day)	High intake ≥81.55(mg/day)						Low intake < 62.59 (mg/day)	High intake ≥62.59(mg/day)					
			Crude			Adjust				Crude			Adjust		
			β	95% CI	*p*	β	95% CI	*p*		β	95% CI	*p*	β	95% CI	*p*
			
Anthropometric factors															
BMI (kg/m^2^)	Low risk	Reference	Reference						Reference	Reference					
	Moderate risk	–	−3.46	−5.44 to −1.49	**<0.001**	−2.56	−4.59 to −0.52	**0.01**	–	0.26	−1.7 to 2.23	0.79	0.43	−1.56 to 2.44	0.66
	High risk	–	−0.91	−3.84 to 2.02	0.54	−0.27	−3.28 to 2.73	0.85	–	−0.66	−3.66 to 2.34	0.66	0.62	−2.45 to 3.69	0.69
WC (cm)	Low risk	Reference	Reference						Reference	Reference					
	Moderate risk	–	−7.43	−12.48 to −2.39	**<0.001**	−5.67	−10.79 to −0.56	**0.03**	–	0.82	−4.17 to 5.81	0.74	2.53	−2.48 to 7.54	0.32
	High risk	–	−0.82	−8.22 to 6.58	0.82	0.65	−6.78 to 8.08	0.86	–	−3.08	−10.67 to 4.5	0.42	1.60	−6.04 to 9.26	0.68
WHR	Low risk	Reference	Reference						Reference	Reference					
	Moderate risk	–	−0.01	−0.043 to 0.013	0.29	<0.001	−0.033 to 0.023	0.71	–	0.00	−0.02 to 0.03	0.85	0.01	−0.014 to 0.041	0.34
	High risk	–	0.02	−0.016 to 0.066	0.23	0.03	−0.007 to 0.075	0.1	–	<0.001	−0.051 to 0.032	0.65	0.01	−0.023 to 0.061	0.37
**Body composition**															
VFL (cm^2^)	Low risk	Reference	Reference						Reference	Reference					
	Moderate risk	–	−2.21	−3.96 to −0.46	**0.01**	−1.61	−3.4 to 0.17	0.07	–	0.10	−1.63 to 1.83	0.91	0.87	−0.87 to 2.63	0.32
	High risk	–	0.57	−2 to 3.15	0.66	1.44	−1.15 to 4.05	0.27	–	−0.00	−2.65 to 2.64	0.99	1.67	−1.01 to 4.36	0.22
OD (%)	Low risk	Reference	Reference						Reference	Reference					
	Moderate risk	–	−13.04	−24.62 to −1.47	**0.02**	−11.20	−22.67 to 0.27	**0.05**	–	−4.03	−15.45 to 7.38	0.48	0.85	−10.48 to 12.18	0.88
	High risk	–	−3.69	−20.75 to 13.36	0.67	−1.25	−17.99 to 15.47	0.88	–	−8.65	−26.06 to 8.76	0.33	−0.90	−18.24 to 16.43	0.91
**Biochemical variables**															
FBS (mg/dL)	Low risk	Reference	Reference						Reference	Reference					
	Moderate risk	–	−0.85	−6.43 to 4.72	0.76	0.07	−5.82 to 5.97	0.98	–	−0.09	−5.53 to 5.34	0.97	0.86	−4.82 to 6.56	0.76
	High risk	–	−3.58	−11.92 to 4.75	0.4	−2.92	−11.71 to 5.86	0.51	–	−0.76	−9.2 to 7.68	0.86	−0.12	−9.15 to 8.91	0.97
TC (mg/dL)	Low risk	Reference	Reference						Reference	Reference					
	Moderate risk	–	−13.03	−34.14 to 8.08	0.22	−7.61	−29.5 to 14.26	0.49	–	−3.11	−23.63 to 17.39	0.76	2.69	−18.31 to 23.71	0.8
	High risk	–	−16.48	−48.02 to 15.06	0.3	−4.17	−36.79 to 28.43	0.8	–	−10.82	−42.67 to 21.02	0.5	−11.42	−44.78 to 21.93	0.5
TG (mg/dL)	Low risk	Reference	Reference						Reference	Reference					
	Moderate risk	–	−5.96	−41.06 to 29.12	0.73	2.96	−34.27 to 40.21	0.87	–	4.04	−30.93 to 39.01	0.82	9.17	−27.45 to 45.79	0.62
	High risk	–	−84.30	−137.58 to −31.02	**<0.001**	−79.11	−135.52 to −22.69	**<0.001**	–	8.23	−47.21 to 63.68	0.77	15.75	−44.36 to 75.86	0.6
HDL (mg/dL)	Low risk	Reference	Reference						Reference	Reference					
	Moderate risk	–	1.75	−4.72 to 8.22	0.59	3.94	−2.82 to 10.71	0.25	–	−4.03	−10.35 to 2.28	0.21	−2.17	−8.8 to 4.44	0.51
	High risk	–	8.39	−1.28 to 18.06	0.08	12.56	2.47–22.65	**0.01**	–	−7.74	−17.56 to 2.06	0.12	−7.31	−17.82 to 3.19	0.17
LDL (mg/dL)	Low risk	Reference	Reference						Reference	Reference					
	Moderate risk	–	−2.68	−16.77 to 11.39	0.7	3.94	−10.74 to 18.63	0.59	–	0.95	−10.35 to 2.28	0.21	3.46	−8.8 to 4.44	0.51
	High Risk	–	−11.13	−32.18 to 9.91	0.3	−4.83	−26.72 to 17.06	0.66	–	−16.77	−17.56 to 2.06	0.12	−22.00	−17.82 to 3.19	0.17
AST (IU/L)	Low risk	Reference	Reference						Reference	Reference					
	Moderate risk	–	−2.14	−6.57 to 2.28	0.34	−1.86	−6.51 to 2.79	0.43	–	0.70	−3.61 to 5.03	0.74	1.15	−3.36 to 5.67	0.61
	High risk	–	−0.78	−5.83 to 7.4	0.81	2.35	−4.58 to 9.29	0.5	–	−0.79	−7.5 to 5.9	0.81	1.25	−5.91 to 8.42	0.73
ALT (IU/L)	Low risk	Reference	Reference						Reference	Reference					
	Moderate risk	–	−7.02	−14.74 to 0.69	0.07	−5.31	−13.58 to 2.96	0.2	–	0.83	−6.77 to 8.43	0.83	2.34	−5.72 to 10.42	0.56
	High risk	–	3.75	−7.78 to 15.28	0.52	6.04	−6.28 to 18.37	0.33	–	−5.77	−17.58 to 6.02	0.33	−2.46	−15.27 to 10.34	0.7

B, Standard Error; GRS, Genetic risk score; BMI, Body mass index; WC, waist circumference; WHR, waist height ratio; FBS, fasting blood sugar; TG, Triglyceride; LDL, Low density lipoprotein; HDL, High density lipoprotein; OD, Obesity degree; VFL, Visceral fat level.

Flavonoids High intake ≥ 81.55(mg/day), Polyphenol High intake ≥ 62.59(mg/day).

*p*-values < 0.05 are in bold.

Adjust = adjusted for potential confounding factors including (age, IPAC and total energy intake).

Low Risk: 0,1,2 Risk allele, Moderate Risk: 3,4 Risk allele, High Risk: 5,6 Risk allele.

**Figure 3:**
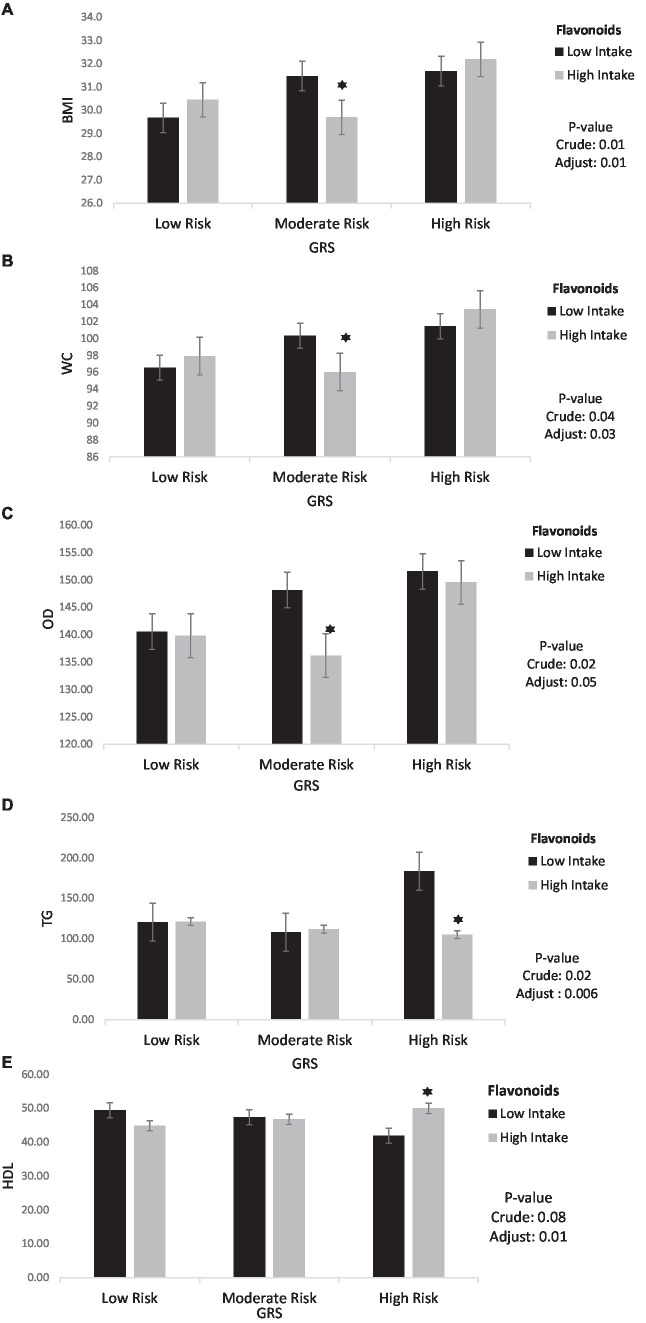
Interaction between flavonoids and GRS on **(A)** BMI, **(B)** WC, **(C)** OD, **(D)** TG, **(E)** HDL. The interaction between low and high intake of Flavonoids and GRS on BMI, WC, OD, TG, HDL. Data shown are mean ± standard error of the mean. BMI, Body mass index; WC, Waist circumference; OD, Obesity degree; TG, Triglycerides; HDL, High density lipoprotein; GRS, genetic risk score. Adjust = adjusted for potential confounding factors including (age, IPAC and total energy intake). The asterisk (*) represents the *p*-value of the statistical test. The asterisk means that the *p*-value is less than 0.05.

The findings also showed that the significant negative interaction between moderate GRS and high intake flavonoids with BMI (95%CI = −5.44, −1.49, *p* < 0.001), WC (95%CI = −12.48, −2.39, *p* < 0.001), VFL (95%CI = −3.96, −0.46, *p* = 0.01) and OD (95%CI = −24.62, −1.47, *p* = 0.02) in the crude model. After adjusting for age, IPAC, and total energy intake in model 1, the interaction between moderate GRS and high intake flavonoids with BMI (95%CI = −4.59, −0.52, *p* = 0.01), WC (95%CI = −10.79, −0.56, *p* = 0.03) and OD (95%CI = −22.67, 0.27, *p* = 0.05) remained negative. Interaction between Flavonoids and GRS on BMI, WC, OD, TG and HDL is shown in Figure 3.

Furthermore, there was no significant interaction found between moderate/high GRS and polyphenol with cardiometabolic risk factors in both the crude and adjusted models.

## Discussion

The purpose of this cross-sectional study was to investigate the relationship between polyphenol consumption and genes (MC4r, Cav-1, and Cry1) and cardiometabolic risk factors in overweight and obese Iranian women.

The findings of our study revealed a significant negative interaction between high GRS and high intake TDPI with FBS in both crude and adjusted models. Also, a significant negative interaction was observed between high GRS and high intake of phenolic acid on TG in the adjusted model. Moreover, a significant negative interaction was observed between high GRS and high intake flavonoids with TG, and a significant positive interaction was observed between high GRS and high intake flavonoids with HDL in the adjusted model. Furthermore, a significant negative interaction between moderate GRS and high intake TDPI on BMI, WC, VLF and FBS levels. According to our results, high intake of TDPI is associated with significant interaction with decreased levels of BMI, WC, VLF, and FBS in participants at moderate risk of GRS. In addition, in this study reported a significant negative interaction between moderate GRS and flavonoid on BMI, WC and OD levels.

However, there was no significant interaction found between moderate/high GRS and high intake stilbenes, polyphenols, and lignans with cardiometabolic risk factors in both the crude and adjusted models. In a study, there was no significant association total polyphenol and stilbenes with FBS, TG, HDL in participants. Also, there was no association between lignans with cholesterol, TG, HDL, DBP (50). The another study after adjusted for age, BMI, physical activity, and total energy intake, there was no association between Cry1 genotypes with cholesterol, TG, HDL, LDL, SBP, DBP (51). There was no association between Cav-1 rs3807992 genotypes with FBS, insulin, TC, TG (52).

Polyphenols are effective in cardiovascular health due to their antioxidant, blood sugar control, anti-inflammatory, and lipid profile control effects (25, 53, 54). In a study, it was discovered that a better diet quality, which might include polyphenol-rich foods, was significantly linked to a reduction in cardiometabolic risk factors (55). Furthermore, a cohort study spanning 10 years and involving more than 450,000 participants across 10 European countries revealed that higher diet quality index (DQI) scores were linked to a reduced risk of CVD mortality and its associated risk factors, such as dyslipidemia and hyperglycemia (56).

GRS play a key role in comprehending various aspects of body homeostasis, especially with respect to conditions such as type 2 diabetes (T2DM) and cardiometabolic risk factors (9, 57). It seems that the effects of MC4R, Cav-1 and Cry1 genes on body composition and metabolic parameters may depend on the quality of the diet (58). Polyphenols have antioxidant and anti-inflammatory properties that can improve the quality of food. Based on the evidence, these compounds have the ability to influence the interaction and interactions between diet, genes, and metabolic parameters (59, 60). Polyphenols and their various types play a role in this interaction by either activating or deactivating genes that are associated with obesity. The precise molecular and cellular mechanism behind this process is still not entirely comprehended (36). Previous research has shown that gene-diet interactions have an impact on metabolic factors, and our study’s findings are consistent with this. A cross-sectional study found a relationship between the dietary inflammatory index (DII) and the rs17782313 mutation, which influences body composition (61). Hianza et al. conducted a study on individuals with a genetic predisposition to obesity and discovered that adopting a healthy diet led to a decrease in risk factors associated with CVD (62).

A study conducted by Aali et al. has significantly validated the findings of our study. Both studies have found a negative correlation between the consumption of polyphenols and its various forms with indicators of body composition (such as WC, WHR, WHtR) and metabolic parameters such as glycemia (FBG, HOMA-IR) and lipids (CHOL, TG). Also, Aali’s study has reported a positive correlation with HDL cholesterol. However, our study did not find any significant correlation between the consumption of lignans and stilbenes with body composition and CVD risk factors, Aali’s study did report such a correlation, which is the only point of difference between the two studies (50).

Numerous studies have confirmed that GRS increases the risk of CVD (9), the exact way in which food compounds like polyphenols can mitigate this effect remains unclear. The statistical power of the analysis can be affected by factors such as sample size, food components used, genetic variations, and gender, which can cause discrepancies in study findings. Evidence shows that polyphenols and its types reduce TG accumulation, increase lipolysis, decrease lipogenesis, and increase energy consumption, which may be an acceptable reason to justify the negative interaction between the consumption of polyphenols and GRS on body composition and some of the risk factors of CVD (63). Polyphenols play a crucial role in regulating blood sugar levels and improving the body’s ability to respond to insulin by decreasing the production of specific hormones (64). Moreover, these compounds have the ability to control the process of lipolysis by activating hormone-sensitive lipase (65). Polyphenols enhance the body’s antioxidant system, reduce fat oxidation, and enhance the activity of antioxidant enzymes (66, 67). Therefore, it is anticipated that enhancing the consumption of a diet rich in polyphenols and their various forms will result in enhanced body composition and a reduction in certain risk factors associated with CVD (68). These results are confirmed by our study.

The present study is one of the first studies to examine the interaction of polyphenol intake with GRS on metabolic parameters, which is one of the strengths of this study. Some limitations of this research include the cross-sectional design of the study, the lack of investigation into causal interactions, the use of memory-based tools such as FFQ, and the inability to generalize the results to men’s gender.

## Conclusion

Our findings indicate that individuals who consume high GRS and polyphenols have a significant negative effect on FBS and TG levels, as well as a significant positive effect on HDL. Therefore, a high intake of polyphenols in individuals with high GRS may have a protective effect on cardiometabolic risk. This finding indicates that the interaction of dietary components, such as polyphenols, with genetic risk factors over cardiometabolic risk factors is of great importance. Further research is necessary in the future to validate this association.

## Data availability statement

The raw data supporting the conclusions of this article will be made available by the authors, without undue reservation.

## Ethics statement

The studies involving humans were approved by the authors and the Ethics Committee of Tehran University of Medical Sciences (IR.TUMS.MEDICINE.REC.1402.636) have received approval for the method and protocol of the current study. The studies were conducted in accordance with the local legislation and institutional requirements. The participants provided their written informed consent to participate in this study.

## Author contributions

ZR: Writing – original draft, Writing – review & editing. AM: Investigation, Validation, Writing – review & editing. FA: Data curation, Formal analysis, Methodology, Software, Writing – review & editing. SD: Conceptualization, Writing – original draft. YA: Methodology, Writing – review & editing. KM: Funding acquisition, Visualization, Writing – review & editing.

## Figures and Tables

**Table:** 

ALT	Alanine aminotransferase
AST	Aspartate aminotransferase
CVD	Cardiovascular diseases
BMI	Body mass index
WC	Waist circumference
DPI	Dietary polyphenols intake
FBS	Fasting blood glucose
FPG	Fasting plasma glucose
MC4R	Melanocortin 4 receptor
Cry1	Cryptochrome 1 gene
CAV1	Caveolin-1
FFQ	Food frequency questionnaire
DQI	diet quality index
PA	Physical activity
IPAQ	International physical activity questionnaire-short form
HC	Hp circumference
WHR	Waist-to-hip ratio
VFL	Visceral fat level
OD	Obesity degree
GOD-PAP	Glucose oxidase-phenol 4-aminoantipyrine peroxidase
GPOPAP	Glycerol-3-phosphate oxidase–phenol 4-aminoantipyrine peroxidase
TG	Triglyceride
HDL	High-density lipoprotein
LDL	Low-density lipoprotein
SBP	Systolic blood pressure
DBP	Diastolic blood pressure
GRS	Genetic risk score
TC	Total cholesterol
TDPI	Total dietary polyphenols index
SLM	Soft lean mass
FFM	Fat-free mass
SMM	Skeletal muscle mass
DII	Dietary inflammatory index

## References

[1] Benjamin EJ Virani SS Callaway CW Chamberlain AM Chang AR Chang S. Correction to: heart disease and stroke Statistics-2018 update: a report from the American Heart Association. Circulation. (2018) 137:e493–2. doi: 10.1161/CIR.0000000000000573, PMID: 29386200

[2] Gaziano TA Bitton A Anand S Abrahams-Gessel S Murphy A. Growing epidemic of coronary heart disease in low-and middle-income countries. Curr Probl Cardiol. (2010) 35:72–115. doi: 10.1016/j.cpcardiol.2009.10.002 20109979 PMC2864143

[3] Roger VL Go AS Lloyd-Jones DM Adams RJ Berry JD Brown TM. Heart disease and stroke statistics—2011 update: a report from the American Heart Association. Circulation. (2011) 123:e18–e209. doi: 10.1161/CIR.0b013e3182009701, PMID: 21160056 PMC4418670

[4] World Health Organization. Global status report on noncommunicable diseases 2014 World Health Organization (2014). Regions: Africa Americas, Europe, Eastern Mediterranean Western Pacific.

[5] Mozaffarian D Benjamin EJ Go AS Arnett DK Blaha MJ Cushman M. AHA statistical update: Heart disease and stroke statistics AHA (2015). 10.1161/CIR.0000000000000152 25520374

[6] Cho L Davis M Elgendy I Epps K Lindley KJ Mehta PK. Summary of updated recommendations for primary prevention of cardiovascular disease in women: JACC state-of-the-art review. J Am Coll Cardiol. (2020) 75:2602–18. doi: 10.1016/j.jacc.2020.03.060, PMID: 32439010 PMC8328156

[7] McMillan DC Sattar N Lean M McArdle CS. Obesity and cancer. BMJ. (2006) 333:1109–11. doi: 10.1136/bmj.39042.565035.BE1, PMID: 17124223 PMC1661751

[8] Must A Spadano J Coakley EH Field AE Colditz G Dietz WH. The disease burden associated with overweight and obesity. JAMA. (1999) 282:1523–9. doi: 10.1001/jama.282.16.1523 10546691

[9] Gholami F Rasaei N Samadi M Yekaninejad MS Keshavarz SA Javdan G. The relationship of genetic risk score with cardiometabolic risk factors: a cross-sectional study. BMC Cardiovasc Disord. (2022) 22:459. doi: 10.1186/s12872-022-02888-z, PMID: 36324080 PMC9632045

[10] Hruby A Hu FB. The epidemiology of obesity: a big picture. PharmacoEconomics. (2015) 33:673–89. doi: 10.1007/s40273-014-0243-x, PMID: 25471927 PMC4859313

[11] Xiao B Velez Edwards DR Lucas A Drivas T Gray K Keating B. Inference of causal relationships between genetic risk factors for cardiometabolic phenotypes and female-specific health conditions. J Am Heart Assoc. (2023) 12:e026561. doi: 10.1161/JAHA.121.026561, PMID: 36846987 PMC10111435

[12] Igo RP Jr Kinzy TG Cooke Bailey JN. Genetic risk scores. Curr Protoc Hum Genet. (2019) 104:e95. doi: 10.1002/cphg.95 31765077 PMC6941594

[13] Rothman DL Stearns SC Shulman RG. Gene expression regulates metabolite homeostasis during the Crabtree effect: Implications for the adaptation and evolution of Metabolism. Proc Natl Acad Sci U S A. (2021) 118:e2014013118. doi: 10.1073/pnas.2014013118 33372135 PMC7814475

[14] Grant SF Bradfield JP Zhang H Wang K Kim CE Annaiah K. Investigation of the locus near MC4R with childhood obesity in Americans of European and African ancestry. Obesity. (2009) 17:1461–5. doi: 10.1038/oby.2009.53, PMID: 19265794 PMC2860794

[15] Schmid PM Heid I Buechler C Steege A Resch M Birner C. Expression of fourteen novel obesity-related genes in Zucker diabetic fatty rats. Cardiovasc Diabetol. (2012) 11:48–11. doi: 10.1186/1475-2840-11-48 22553958 PMC3398851

[16] Cone RD. The central melanocortin system and energy homeostasis. Trends Endocrinol Metab. (1999) 10:211–6. doi: 10.1016/S1043-2760(99)00153-8 10407394

[17] Jee SH Sull JW Park J Lee SY Ohrr H Guallar E. Body-mass index and mortality in Korean men and women. N Engl J Med. (2006) 355:779–87. doi: 10.1056/NEJMoa054017 16926276

[18] Sun Y Sun J Wu J Yang M. Combined effects of FTO rs9939609 and MC4R rs17782313 on elevated nocturnal blood pressure in the Chinese Han population: cardiovascular topics. Cardiovasc J Afr. (2016) 27:21–4. doi: 10.5830/CVJA-2015-064, PMID: 26324055 PMC4816968

[19] Greenfield JR Miller JW Keogh JM Henning E Satterwhite JH Cameron GS. Modulation of blood pressure by central melanocortinergic pathways. N Engl J Med. (2009) 360:44–52. doi: 10.1056/NEJMoa0803085 19092146

[20] de Souza GM de Albuquerque Borborema ME de Lucena TMC da Silva Santos AF de Lima BR de Oliveira DC. Caveolin-1 (CAV-1) up regulation in metabolic syndrome: all roads leading to the same end. Mol Biol Rep. (2020) 47:9245–50. doi: 10.1007/s11033-020-05945-y, PMID: 33123955

[21] Jia G Sowers JR. Caveolin-1 in cardiovascular disease: a double-edged sword. Diabetes. (2015) 64:3645–7. doi: 10.2337/dbi15-0005, PMID: 26494216 PMC4613981

[22] Voghel G Thorin-Trescases N Farhat N Mamarbachi M Villeneuve L Perrault LP. Replicative senescence of vascular endothelial cells isolated from coronary patients is worsened by oxidative stress associated with risk factors for cardiovascular disease. FASEB J. (2008) 22. doi: 10.1096/fasebj.22.1_supplement.964.24

[23] Fourier C Ran C Sjöstrand C Waldenlind E Steinberg A Belin AC. The molecular clock gene cryptochrome 1 (CRY1) and its role in cluster headache. Cephalalgia. (2021) 41:1374–81. doi: 10.1177/03331024211024165, PMID: 34256648 PMC8592106

[24] Ye D Cai S Jiang X Ding Y Chen K Fan C. Associations of polymorphisms in circadian genes with abdominal obesity in Chinese adult population. Obes Res Clin Pract. (2016) 10:S133–41. doi: 10.1016/j.orcp.2016.02.002, PMID: 26923944

[25] Lanuza F Zamora-Ros R Bondonno NP Meroño T Rostgaard-Hansen AL Riccardi G. Dietary polyphenols, metabolic syndrome and cardiometabolic risk factors: an observational study based on the DCH-NG subcohort. Nutr Metab Cardiovasc Dis. (2023) 33:1167–78. doi: 10.1016/j.numecd.2023.02.022, PMID: 36948936

[26] Lemmer B Oster H. The role of circadian rhythms in the hypertension of diabetes mellitus and the metabolic syndrome. Curr Hypertens Rep. (2018) 20:1–9. doi: 10.1007/s11906-018-0843-5 29730779

[27] Dashti HS Smith CE Lee YC Parnell LD Lai CQ Arnett DK. CRY1 circadian gene variant interacts with carbohydrate intake for insulin resistance in two independent populations: Mediterranean and north American. Chronobiol Int. (2014) 31:660–7. doi: 10.3109/07420528.2014.886587, PMID: 24548145 PMC4041822

[28] Mirzababaei A Daneshzad E Shiraseb F Pourreza S Setayesh L Clark CCT. Variants of the cry 1 gene may influence the effect of fat intake on resting metabolic rate in women with overweight of obesity: a cross-sectional study. BMC Endocr Disord. (2021) 21:196. doi: 10.1186/s12902-021-00860-0, PMID: 34610814 PMC8493740

[29] Gholami F Samadi M Rasaei N Yekaninejad MS Keshavarz SA Javdan G. Interactions between genetic risk score and healthy plant diet index on Cardiometabolic risk factors among obese and overweight women. Clin Nutr Res. (2023) 12:199–217. doi: 10.7762/cnr.2023.12.3.199 37593209 PMC10432161

[30] Adriouch S Lampuré A Nechba A Baudry J Assmann K Kesse-Guyot E. Prospective association between total and specific dietary polyphenol intakes and cardiovascular disease risk in the Nutrinet-Santé French cohort. Nutrients. (2018) 10:1587. doi: 10.3390/nu10111587, PMID: 30380657 PMC6266343

[31] Ponzo V Goitre I Fadda M Gambino R de Francesco A Soldati L. Dietary flavonoid intake and cardiovascular risk: a population-based cohort study. J Transl Med. (2015) 13:1–13. doi: 10.1186/s12967-015-0573-2 26152229 PMC4494724

[32] Pandey KB Rizvi SI. Plant polyphenols as dietary antioxidants in human health and disease. Oxidative Med Cell Longev. (2009) 2:270–8. doi: 10.4161/oxim.2.5.9498, PMID: 20716914 PMC2835915

[33] Aloo SO Ofosu FK Kim NH Kilonzi SM Oh DH. Insights on dietary polyphenols as agents against metabolic disorders: obesity as a target disease. Antioxidants (Basel). (2023) 12. doi: 10.3390/antiox12020416 PMC9952395 36829976

[34] Adriouch S Kesse-Guyot E Feuillet T Touvier M Olié V Andreeva V. Total and specific dietary polyphenol intakes and 6-year anthropometric changes in a middle-aged general population cohort. Int J Obes. (2018) 42:310–7. doi: 10.1038/ijo.2017.227, PMID: 28928462

[35] Zujko ME Waśkiewicz A Witkowska AM Szcześniewska D Zdrojewski T Kozakiewicz K. Dietary total antioxidant capacity and dietary polyphenol intake and prevalence of metabolic syndrome in polish adults: a nationwide study. Oxidative Med Cell Longev. (2018) 2018:1–10. doi: 10.1155/2018/7487816, PMID: 29770169 PMC5892227

[36] Grosso G Stepaniak U Micek A Stefler D Bobak M Pająk A. Dietary polyphenols are inversely associated with metabolic syndrome in polish adults of the HAPIEE study. Eur J Nutr. (2017) 56:1409–20. doi: 10.1007/s00394-016-1187-z, PMID: 26913852 PMC5486632

[37] Rezazadeh A Omidvar N Tucker KL. Food frequency questionnaires developed and validated in Iran: a systematic review. Epidemiol Health. (2020) 42:e2020015. doi: 10.4178/epih.e2020015 32229793 PMC7340615

[38] Ghaffarpour M. Houshiar-Rad A. Kianfar H., The manual for household measures, cooking yields factors and edible portion of foods. Tehran: Nashre Olume Keshavarzy, (1999). 7: p. 42–58.

[39] Neveu V Perez-Jimenez J Vos F Crespy V du Chaffaut L Mennen L. Phenol-explorer: an online comprehensive database on polyphenol contents in foods. Database. (2010) 2010:bap024. doi: 10.1093/database/bap024 20428313 PMC2860900

[40] Fidanza F. Nutritional status assessment: A manual for population studies. United kingdom: Springer (2013).

[41] Yarizadeh HSL Roberts C Yekaninejad MS Mirzaei K. Nutrient pattern of unsaturated fatty acids and vitamin E increase resting metabolic rate of overweight and obese women. Int J Vitam Nutr Res. (2022) 92:214–22. doi: 10.1024/0300-9831/a000664, PMID: 32672509

[42] Penumarthy S Penmetsa GS Mannem S. Assessment of serum levels of triglycerides, total cholesterol, high-density lipoprotein cholesterol, and low-density lipoprotein cholesterol in periodontitis patients. J Indian Soc Periodontol. (2013) 17:30–5. doi: 10.4103/0972-124X.107471, PMID: 23633769 PMC3636940

[43] MWer S Dykes D Polesky H. A simple salting out procedure for extracting DNA from human nucleated cells. Nucleic Acids Res. (1988) 16:1215. doi: 10.1093/nar/16.3.1215 3344216 PMC334765

[44] Myakishev MV Khripin Y Hu S Hamer DH. High-throughput SNP genotyping by allele-specific PCR with universal energy-transfer-labeled primers. Genome Res. (2001) 11:163–9. doi: 10.1101/gr.157901, PMID: 11156625 PMC311033

[45] Abaj F Koohdani F Rafiee M Alvandi E Yekaninejad MS Mirzaei K. Interactions between Caveolin-1 (rs3807992) polymorphism and major dietary patterns on cardio-metabolic risk factors among obese and overweight women. BMC Endocr Disord. (2021) 21:138. doi: 10.1186/s12902-021-00800-y, PMID: 34210318 PMC8247154

[46] Yu K Li L Zhang L Guo L Wang C. Association between MC4R rs17782313 genotype and obesity: a meta-analysis. Gene. (2020) 733:144372. doi: 10.1016/j.gene.2020.144372, PMID: 31954858

[47] Tangestani H Emamat H Yekaninejad MS Keshavarz SA Mirzaei K. Variants in circadian rhythm gene Cry1 interacts with healthy dietary pattern for serum leptin levels: a cross-sectional study. Clin Nutr Res. (2021) 10:48–58. doi: 10.7762/cnr.2021.10.1.48, PMID: 33564652 PMC7850819

[48] Miranda AM Steluti J Norde MM Fisberg RM Marchioni DM. The association between genetic risk score and blood pressure is modified by coffee consumption: gene–diet interaction analysis in a population-based study. Clin Nutr. (2019) 38:1721–8. doi: 10.1016/j.clnu.2018.07.033, PMID: 30119983

[49] Ainsworth BE Haskell WL Whitt MC Irwin ML Swartz AM Strath SJ. Compendium of physical activities: an update of activity codes and MET intensities. Med Sci Sports Exerc. (2000) 32:S498–516. doi: 10.1097/00005768-200009001-00009, PMID: 10993420

[50] Aali Y Ebrahimi S Shiraseb F Mirzaei K. The association between dietary polyphenol intake and cardiometabolic factors in overweight and obese women: a cross-sectional study. BMC Endocr Disord. (2022) 22:120. doi: 10.1186/s12902-022-01025-3, PMID: 35538570 PMC9088119

[51] Firouzabadi FD Mirzababaei A Shiraseb F Tangestani H Mirzaei K. The interaction between CRY1 polymorphism and alternative healthy eating index (AHEI) on cardiovascular risk factors in overweight women and women with obesity: a cross-sectional study. BMC Endocr Disord. (2023) 23:172. doi: 10.1186/s12902-023-01429-9, PMID: 37580741 PMC10424458

[52] Abaj F Mirzababaei A Hosseininasab D Bahrampour N Clark CCT Mirzaei K. Interactions between Caveolin-1 polymorphism and plant-based dietary index on metabolic and inflammatory markers among women with obesity. Sci Rep. (2022) 12:9088. doi: 10.1038/s41598-022-12913-y, PMID: 35641515 PMC9156773

[53] Haș IM Teleky BE Vodnar DC Ștefănescu BE Tit DM Nițescu M. Polyphenols and Cardiometabolic health: knowledge and concern among Romanian people. Nutrients. (2023) 15:2281. doi: 10.3390/nu15102281, PMID: 37242164 PMC10221773

[54] Wang S du Q Meng X Zhang Y. Natural polyphenols: a potential prevention and treatment strategy for metabolic syndrome. Food Funct. (2022) 13:9734–53. doi: 10.1039/D2FO01552H 36134531

[55] Mohamadi A Shiraseb F Mirzababaei A AkbarySedigh A Ghorbani M Clark CCT. The association between adherence to diet quality index and cardiometabolic risk factors in overweight and obese women: a cross-sectional study. Front Public Health. (2023) 11:11. doi: 10.3389/fpubh.2023.1169398 PMC10374417 37521997

[56] Lassale C Gunter MJ Romaguera D Peelen LM van der Schouw YT Beulens JWJ. Diet quality scores and prediction of all-cause, cardiovascular and cancer mortality in a pan-European cohort study. PLoS One. (2016) 11:e0159025. doi: 10.1371/journal.pone.0159025, PMID: 27409582 PMC4943719

[57] Norris JM Rich SS. Genetics of glucose homeostasis: implications for insulin resistance and metabolic syndrome. Arterioscler Thromb Vasc Biol. (2012) 32:2091–6. doi: 10.1161/ATVBAHA.112.255463, PMID: 22895670 PMC3988457

[58] Hosseininasab D Mirzababaei A Abaj F Firoozi R Clark CCT Mirzaei K. Are there any interactions between modified Nordic-style diet score and MC4R polymorphism on cardiovascular risk factors among overweight and obese women? A cross-sectional study. BMC Endocr Disord. (2022) 22:221. doi: 10.1186/s12902-022-01132-1, PMID: 36050672 PMC9434967

[59] Barth SW Koch TCL Watzl B Dietrich H Will F Bub A. Moderate effects of apple juice consumption on obesity-related markers in obese men: impact of diet–gene interaction on body fat content. Eur J Nutr. (2012) 51:841–50. doi: 10.1007/s00394-011-0264-6, PMID: 22038464

[60] Soyalan B Minn J Schmitz HJ Schrenk D Will F Dietrich H. Apple juice intervention modulates expression of ARE-dependent genes in rat colon and liver. Eur J Nutr. (2011) 50:135–43. doi: 10.1007/s00394-010-0124-9, PMID: 20652274

[61] Yarizadeh H Mirzababaei A Ghodoosi N Pooyan S Djafarian K Clark CCT. The interaction between the dietary inflammatory index and MC4R gene variants on cardiovascular risk factors. Clin Nutr. (2021) 40:488–95. doi: 10.1016/j.clnu.2020.04.044 32586686

[62] Heianza Y Zhou T Sun D Hu FB Qi L. Healthful plant-based dietary patterns, genetic risk of obesity, and cardiovascular risk in the UK biobank study. Clin Nutr. (2021) 40:4694–701. doi: 10.1016/j.clnu.2021.06.018, PMID: 34237696 PMC8338907

[63] Boccellino M D’Angelo S. Anti-obesity effects of polyphenol intake: current status and future possibilities. Int J Mol Sci. (2020) 21:5642. doi: 10.3390/ijms21165642, PMID: 32781724 PMC7460589

[64] Dao T-MA Waget A Klopp P Serino M Vachoux C Pechere L. Resveratrol increases glucose induced GLP-1 secretion in mice: a mechanism which contributes to the glycemic control. PLoS One. (2011) 6:e20700. doi: 10.1371/journal.pone.0020700, PMID: 21673955 PMC3108962

[65] Nakazato K Song H Waga T. Effects of dietary apple polyphenol on adipose tissues weights in Wistar rats. Exp Anim. (2006) 55:383–9. doi: 10.1538/expanim.55.383 16880686

[66] Dembinska-Kiec A Mykkänen O Kiec-Wilk B Mykkänen H. Antioxidant phytochemicals against type 2 diabetes. Br J Nutr. (2008) 99:ES109. doi: 10.1017/S000711450896579X 18503731

[67] Crespy V Williamson G. A review of the health effects of green tea catechins in in vivo animal models. J Nutr. (2004) 134:3431S–40S. doi: 10.1093/jn/134.12.3431S 15570050

[68] Wang S Moustaid-Moussa N Chen L Mo H Shastri A Su R. Novel insights of dietary polyphenols and obesity. J Nutr Biochem. (2014) 25:1–18. doi: 10.1016/j.jnutbio.2013.09.001, PMID: 24314860 PMC3926750

